# Supplementary material for: PL1 Peptide Engages Acidic Surfaces on Tumor-Associated Fibronectin and Tenascin Isoforms to Trigger Cellular Uptake
Source: Pharmaceutics. 2021 Nov 24;13(12):1998. doi: 10.3390/pharmaceutics13121998 (PMC8707168; doi:10.3390/pharmaceutics13121998)
Supplement: Supplementary file 1 [file pharmaceutics-13-01998-s001.zip › pharmaceutics-1408444-supplementary.pdf]

pharmaceutics

Supplementary Materials: PL1 Peptide Engages Acidic Surfaces on Tumor-Associated Fibronectin and Tenascin Isoforms to Trigger Cellular Uptake

Prakash Lingasamy; Kristina Põšnograjeva, Sergei Kopanchuk, Allan Tobi, Ago Rinken, Ignacio J. General, Eliana K. Asciutto, Tambet Teesalu

Fibronectin EDB domain (Fn-EDB): 112 a.a. and MW=12008.62 Da
MGSSHHHHHHSSGLVPRGSHMEVPQLTDLSFVDITDSSIGLRWTPLNSSTIIGYRITVVAAGEGIPIFEDFVDSSVGYY
TVTGLEPGIDYDISVITLINGGESAPTTLTQQT

Mutate D52>N56; D49>N49, D12>N12:
MGSSHHHHHHSSGLVPRGSHMEVPQLTDLSFVNITDSSIGLRWTPLNSSTIIGYRITVVAAGEGIPIFENFVNSSVGYY
TVTGLEPGIDYDISVITLINGGESAPTTLTQQT

Mutate D68>N68; D70>N70
MGSSHHHHHHSSGLVPRGSHMEVPQLTDLSFVDITDSSIGLRWTPLNSSTIIGYRITVVAAGEGIPIFEDFVDSSVGYY
TVTGLEPGINYNISVITLINGGESAPTTLTQQT

Mutate E42>Q42; D68>N68, E64>Q64
MGSSHHHHHHSSGLVPRGSHMEVPQLTDLSFVDITDSSIGLRWTPLNSSTIIGYRITVVAAGQGIPIFEDFVDSSVGY
YTVTGLQPGINYDISVITLINGGESAPTTLTQQT

>Tenascin C FnIII C domain (TNC-C) : 112 a.a. and MW=12299.19 Da
MGSSHHHHHHSSGLVPRGSHMEALPLLENLTISDINPYGFTVSWMASENAFDSFLVTVVDSGKLLDPQEFTLSGTQ
RKLELRGLITGIGYEVMVSGFTQGHQTKPLRAEIVT

mutate D13>N13, E70>Q70, E88>Q88
MGSSHHHHHHSSGLVPRGSHMEALPLLENLTISDINPYGFTVSWMASENAFDSFLVTVVDSGKLLDPQEFTLSGTQ
RKLELRGLITGIGYEVMVSGFTQGHQTKPLRAEIVT

mutate D39>N39, D45>N45
MGSSHHHHHHSSGLVPRGSHMEALPLLENLTISDINPYGFTVSWMASENAFDSFLVTVVDSGKLLDPQEFTLSGTQ
RKLELRGLITGIGYEVMVSGFTQGHQTKPLRAEIVT

mutate E48>Q48
MGSSHHHHHHSSGLVPRGSHMEALPLLENLTISDINPYGFTVSWMASENAFDSFLVTVVDSGKLLDPQEFTLSGTQ
RKLELRGLITGIGYEVMVSGFTQGHQTKPLRAEIVT

Figure S1. The recombinant FN-EDB and TNC-C WT and mutant protein sequences. The primary sequence of WT proteins (FN-EDB and TNC-C) and respective mutant proteins FnEDB (D68N D70N, D52N D49N D12N, and E42Q D68N E64Q) and TNC-C (E48Q, D39N D45N, and D13N E70Q E88Q). The neutral charge amino acid substitutions are shown in red color.

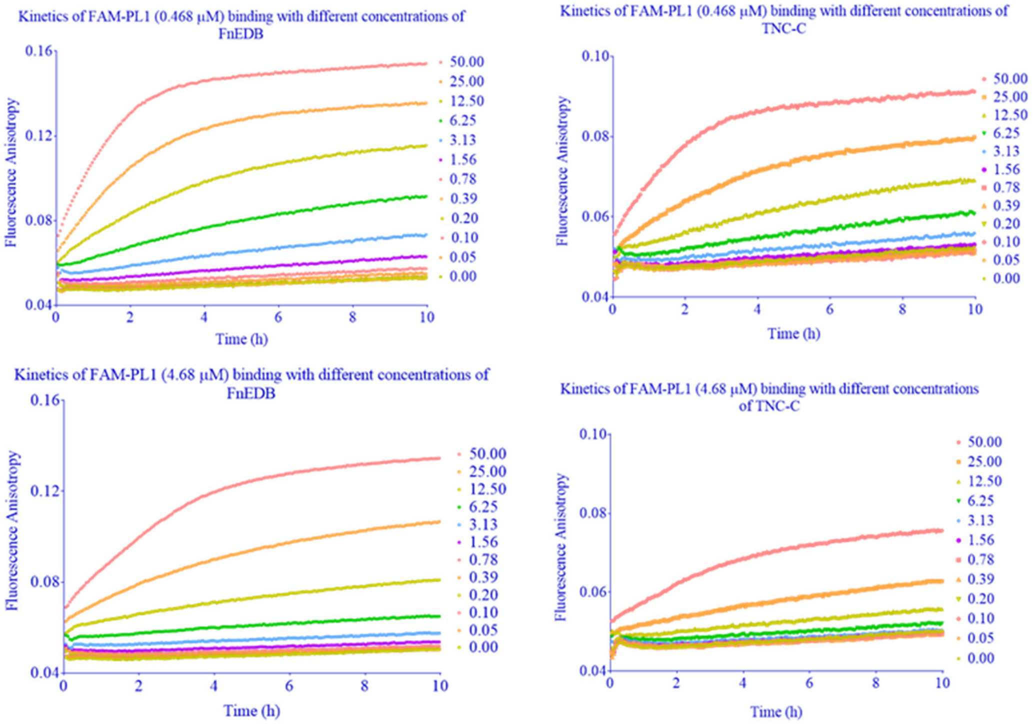

Figure S2. . Fluorescence polarization (FP) saturation binding curves. The time course of FA changes caused by FAM-PL1 to different concentrations of FN-EDB and TNC-C. The receptors were prepared from 50 μM to 0 μM per well in the presence of non-specific binding or absence of total binding to 0. 48 μM and 4.68 μM of FAM-labeled PL1 peptide. According to Eq, at the indicated time points, fluorescence intensity was measured, and anisotropy values were calculated. The data representative of experiments from at least three independent experiments.

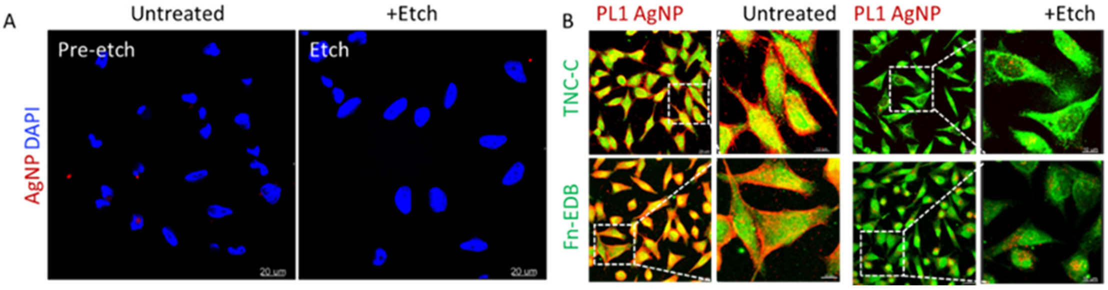

Figure S3. PL1-functionalized silver nanoparticles (AgNPs) colocalize with receptors. (A) Control biotin-AgNPs showed negative binding and internalization in U87-MG cells. Scale bars: 20 μm. (B) The pre-etched PL1-AgNPs (red) colocalize with receptors TNC-C (ScFV G11: Green) and FN-EDB (ScFV L19: Green) Scale bars: 10 μm; magnified images: 5 μm.

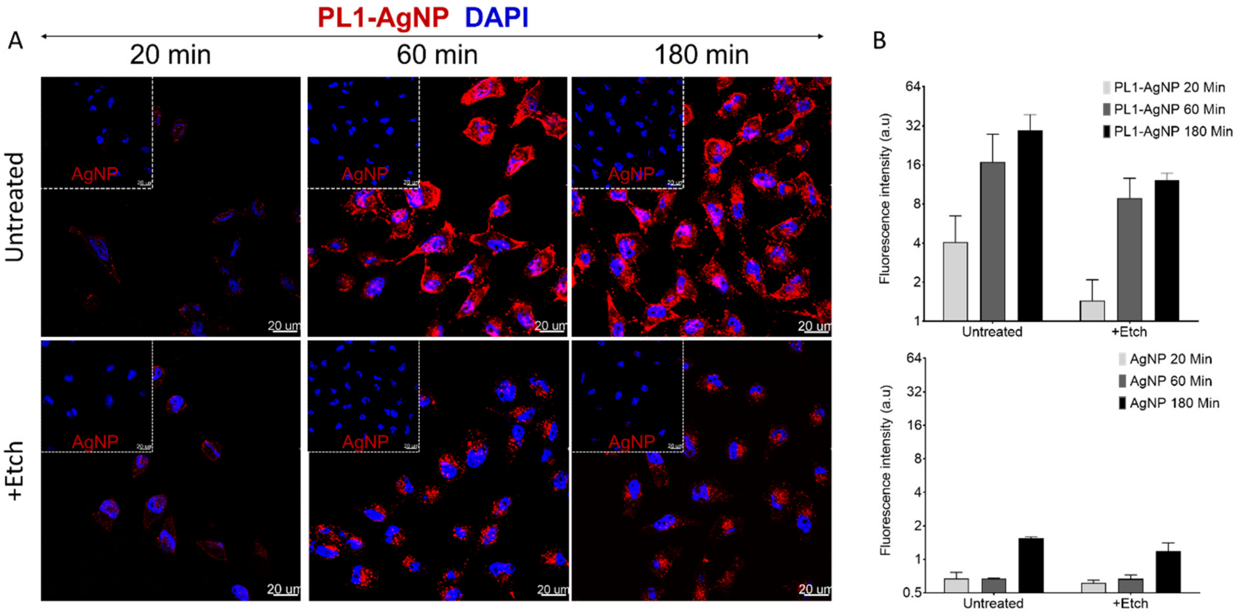

Figure S4. Time-dependent internalization of PL1-functionalized silver nanoparticles (AgNPs) into cells. (A) Confocal microscopy pictures of U87-MG attached cells incubated with PL1-AgNP or AgNP for 20, 60 and 180 min. Some of the cells were etched at 20, 60 and 180 min. The control AgNP samples are completely negative (insets). The pre-etched and etched PL1-AgNPs (red) show superior binding and internalization in a time-dependent manner. (B) Binding and internalization into cells was quantified based on CF555 signal from panel (A) in representative images (mean fluorescence intensity). Error bars: mean ± SD (N=three independent experiments). Scale bars: 20 µm.

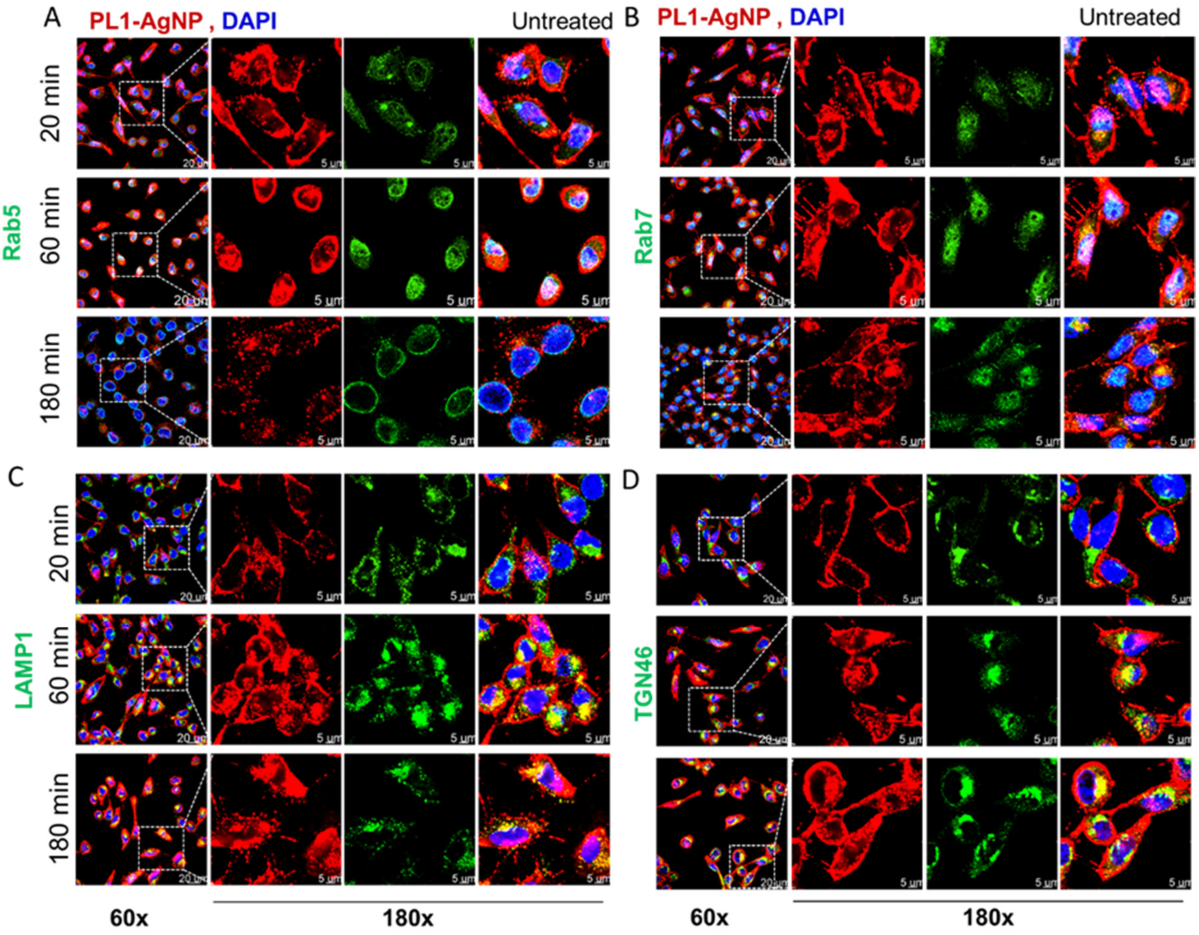

Figure S5. . PL1-functionalized silver nanoparticles (AgNPs) colocalization of endocytic markers in U87-MG cells. Confocal microscopy images of U87-MG attached cells incubated with PL1-AgNPs (red) at 20, 60 and 180 min time points. The cells were stained with (A) early endosome marker Rab 5A (green), (B) late endosome marker Rab 7A (green), (C) lysosome marker LAMP-1 (CD107a) (green), and (D) trans-Golgi network marker TGN46 (green). The cells were counterstained with DAPI (blue). Scale bar: 20 μm (60x); magnified images: 5 μm (180x). Representative images from three independent experiments.
